# Improved Efficiency and Lifetime of Deep‐Blue Hyperfluorescent Organic Light‐Emitting Diode using Pt(II) Complex as Phosphorescent Sensitizer

**DOI:** 10.1002/advs.202100586

**Published:** 2021-06-16

**Authors:** Sungho Nam, Ji Whan Kim, Hye Jin Bae, Yusuke Makida Maruyama, Daun Jeong, Joonghyuk Kim, Jong Soo Kim, Won‐Joon Son, Hyein Jeong, Jaesang Lee, Soo‐Ghang Ihn, Hyeonho Choi

**Affiliations:** ^1^ Samsung Advanced Institute of Technology Samsung Electronics Co., Ltd. 130 Samsung‐ro Suwon‐si Gyeonggi‐do 16678 Republic of Korea; ^2^ Data and Information Technology Center Samsung Electronics Co., Ltd. 1 Samsungjeonja‐ro Hwaseong‐si Gyeonggi‐do 18448 Republic of Korea; ^3^ Display Research Center Samsung Display Co. 1 Samsung‐ro Yongin‐si Gyeonggi‐do 17113 Republic of Korea; ^4^ Department of Electrical and Computer Engineering Inter‐University Semiconductor Research Center Seoul National University Seoul 08826 Republic of Korea

**Keywords:** energy transfer, fluorophores, hyperfluorescent organic light‐emitting diodes, multiple resonance, Pt(II) complex

## Abstract

Although the organic light‐emitting diode (OLED) has been successfully commercialized, the development of deep‐blue OLEDs with high efficiency and long lifetime remains a challenge. Here, a novel hyperfluorescent OLED that incorporates the Pt(II) complex (PtON7‐dtb) as a phosphorescent sensitizer and a hydrocarbon‐based and multiple resonance‐based fluorophore as an emitter (TBPDP and *ν*‐DABNA) in the device emissive layer (EML), is proposed. Such an EML system can promote efficient energy transfer from the triplet excited states of the sensitizer to the singlet excited states of the fluorophore, thus significantly improving the efficiency and lifetime of the device. As a result, a deep‐blue hyperfluorescent OLED using a multiple resonance‐based fluorophore (*ν*‐DABNA) with Commission Internationale de L'Eclairage chromaticity coordinate *y* below 0.1 is demonstrated, which attains a narrow full width at half maximum of ≈17 nm, fourfold increased maximum current efficiency of 48.9 cd A^−1^, and 19‐fold improved half‐lifetime of 253.8 h at 1000 cd m^−2^ compared to a conventional phosphorescent OLED. The findings can lead to better understanding of the hyperfluorescent OLEDs with high performance.

## Graphical abstract

A novel technology of deep‐blue hyperfluorescent organic light‐emitting diode using Pt(II) complex as phosphorescent sensitizer and multiple resonance‐based fluorophore can pave the way of commercialization.

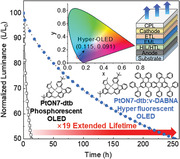

## 1 Introduction

Deep‐blue phosphorescent and thermally activated delayed fluorescent organic light‐emitting diodes (Ph‐OLED and TADF‐OLED), achieving nearly 100% internal quantum efficiency, have been continuously reported.^[^ ^1^, ^2^, ^3^, ^4^ ^]^ The high efficiency of such OLEDs can be attributed to the comprising luminescent materials that utilize both singlet and triplet excitons for emission. However, the operational lifetime of deep‐blue Ph‐ and TADF‐OLEDs remains insufficiently short, preventing their practical use in display and lighting applications.^[^ ^5^, ^6^ ^]^

Device degradation becomes intensified due to the long decay time of the excitons, which can trigger bimolecular annihilations such as triplet–triplet annihilation (TTA) and triplet–polaron quenching (TPQ) in the emissive layer (EML) in the device.^[^ ^5^, ^6^, ^7^, ^8^, ^9^, ^10^, ^11^, ^12^, ^13^, ^14^, ^15^ ^]^ Such TTA and TPQ in deep blue OLEDs generate highly‐energetic excited states (≥6.0 eV) that may highly likely induce chemical bond dissociation in the charge transport/exciton blocking layer and EML, resulting in a permanent decrease in device luminance.^[^ ^5^, ^11^, ^16^, ^17^, ^18^ ^]^ Ensuring short exciton decay time and managing the exciton distribution in the EML is, therefore, of great importance to realize both long lifetime and high efficiency of deep blue OLEDs.^[^ ^11^, ^19^, ^20^, ^21^ ^]^

The combination of fluorescent emitters with the sensitizing materials (or sensitizers) in the OLEDs, namely hyperfluorescent OLED (hyper‐OLED) technology, has been proposed to achieve high efficiency along with a long operational lifetime.^[^ ^22^, ^23^, ^24^, ^25^, ^26^, ^27^, ^28^, ^29^, ^30^, ^31^, ^32^ ^]^ The hyper‐OLED can recycle triplet excitons for emission, which otherwise get lost non‐radiatively, via energy transfer from sensitizers to fluorescent emitters. For example, a Förster resonant energy transfer (FRET) from the T_1_ state of the phosphorescent sensitizer to the S_1_ state of fluorescent emitters was demonstrated.^[^ ^22^ ^]^ Since then, a significant breakthrough in hyper‐OLEDs has been followed by using the TADF sensitizer combined with the conventional red, green, yellow, and blue fluorescent emitters.^[^ ^23^ ^]^ Recently, deep blue Ir(III) phosphor‐ and TADF‐sensitized hyper‐OLEDs using conventional hydrocarbon‐based fluorescent (HCF) emitters were reported. Such devices have enhanced external quantum efficiency (EQE) of up to 19% and improved operational lifetime of up to T80 at 1000 cd m^−2^ ≈320 h (here, we refer to TX as the time during which the luminance decreases to X% of its initial luminance).^[^ ^33^, ^34^, ^35^, ^36^, ^37^ ^]^ Deep blue hyper‐OLEDs incorporating multiple resonance fluorescent (MRF) emitters have been spotlighted, achieving superior luminescent properties such as high photoluminescence quantum yield (PLQY) of > 0.8, small Stokes shift (≈40 nm), and narrow full width at half maximum (FWHM) of < 30 nm.^[^ ^38^, ^39^, ^40^, ^41^, ^42^, ^43^, ^44^, ^45^ ^]^ Particularly, a recent deep‐blue hyper‐OLED that employed a TADF sensitizer with a high reverse intersystem crossing rate (k_RISC_ = 2.36 × 10^6^ s^−1^) and an MRF emitter is noteworthy.^[^ ^46^ ^]^ It exhibited the extremely high maximum external quantum efficiency (EQE_max_) of 32.5% and long device lifetime of T80 at 1000 cd m^−2^ > 60 h with FWHM of ≈29 nm. Very recently, two‐unit stacked tandem hyper‐OLED exhibited the good stability and high efficiency (EQE of 32% and T95 of 18 h at 1000 cd m^−2^) as well as narrow emission with FWHM of ≈19 nm.^[^ ^47^ ^]^ Despite these efforts, most of the hyper‐OLEDs reported so far suffered from an inefficient energy transfer from a sensitizer to an emitter, rendering it hard to achieve highly saturated emission in deep blue.

In this work, we present deep‐blue hyper‐OLEDs that incorporate a Pt(II) complex as a sensitizer and either a HCF or MRF emitter in the EML. A sensitizer and an emitter were carefully chosen such that they have a large spectral overlap between the photoluminescence (PL) of the sensitizer and the absorption of the emitter to enhance FRET, as well as direct exciton formation on the sensitizer. The emission spectrum of the hyper‐OLED coincides with that of the conventional fluorescent OLED due to the efficient FRET from the sensitizer to the HCF or MRF emitters, confirmed by the steady‐state and transient PL spectroscopic measurements. We demonstrate the highly efficient and stable deep‐blue hyper‐OLEDs using the Pt‐sensitizer, regardless of the emitter type (i.e., HCF or MRF). In particular, the top‐emission hyper‐OLEDs with the MRF emitter exhibited a deep‐blue emission with Commission Internationale de L'Eclairage (CIE 1931) chromaticity coordinates of (0.115, 0.091) along with 3.6‐fold improved maximum current efficiency of 48.9 cd A^−1^ and approximately 19‐fold improved T50 at 1000 cd m^−2^ of 253.8 h when compared with those of the conventional phosphorescent OLED without the MRF emitter.

## 2 Results and Discussion

### 2.1 Key Requirements for Hyper‐OLEDs

**Figure 1:**
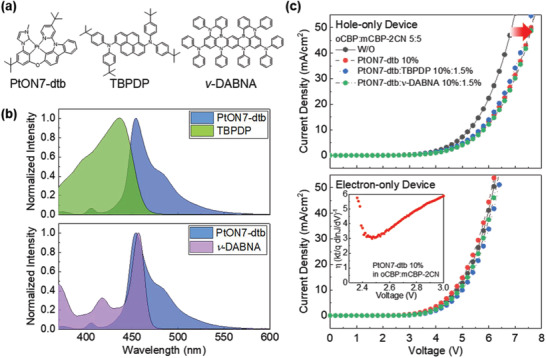
a) Chemical structures of PtON7‐dtb Ph‐sensitizer, and TBPDP and *ν*‐DABNA emitters. b) Spectral overlap region between PtON7‐dtb photoluminescence (PL) and absorption spectra of TBPDP and *ν*‐DABNA emitters in toluene (10^−5^ m solution). c) Current density–voltage (*J–V*) curves of hole‐ and electron‐only devices (HOD) for oCBP:mCBP‐2CN co‐host only, PtON7‐dtb 10%, PtON7‐dtb:TBPDP 10%:1.5%, and PtON7‐dtb:*ν*‐DABNA 10%:1.5% in oCBP:mCBP‐2CN (5:5) matrix. Inset shows the ideality factor (*η*)‐V characteristics of PtON7‐dtb 10% in oCBP:mCBP‐2CN (5:5) OLED device.

The chemical structures of the PtON7‐dtb used as a sensitizer and HCF (N^1^,N^1^,N^6^,N^6^‐tetrakis(4‐(tert‐butyl)phenyl)pyrene‐1,6‐diamine, TBPDP) and MRF (N^7^,N^7^,N^13^,N^13^,5,9,11,15‐octaphenyl‐5,9,11,15‐tetrahydro‐5,9,11,15‐tetraaza‐19b,20b‐diboradinaphtho[3,2,1‐de:1′,2′,3′‐jk]pent‐acene‐7,13‐diamine, *ν*‐DABNA) emitters are shown in **Figure** **1**a.^[^ ^33^, ^45^, ^48^ ^]^ Hyper‐OLEDs impose certain key requirements: (i) keeping the sufficient distance between the sensitizer and the emitter to suppress their exchange of triplet excitons via Dexter energy transfer. This may be achieved by employing bulky groups such as methyl, tert‐butyl, phenyl, or cumyl in the materials and by introducing low concentrations of the emitters. (ii) maximizing the spectral overlap between the PL of the sensitizer and the absorption of the emitter to enhance an efficiency of FRET, and (iii) alignment of molecular orbital levels for hosts, sensitizers, and fluorophores to prevent direct exciton formation by charge trapping on the fluorophore.^[^ ^26^, ^27^, ^34^, ^49^ ^]^

To fulfill the requirement (i), we employed a TBPDP emitter with the tert‐butyl group, which can spatially modulate the sensitizer‐to‐emitter distance, and kept doping concentrations of the emitters low in the range of 0.5 to 3%. For the requirement (ii), we obtained large spectral overlap between the PL of PtON7‐dtb and the absorption of the emitters in a solution (see Figure 1b).^[^ ^33^, ^48^, ^49^ ^]^ For the requirement (iii), the sensitizer (PtON7‐dtb) has a comparable highest occupied molecular orbital (HOMO) level to those of the emitters (TBPDP and *ν*‐DABNA). Thus, the sensitizer effectively alleviates the direct charge trapping on the emitters (see Figure S1, Supporting Information). Based on the current density–voltage (*J–V*) curves for hole‐ and electron‐only devices (HOD and EOD) shown in Figure 1c, the injected holes are primarily captured by the sensitizer even in the presence of the TBPDP and *ν*‐DABNA emitters, leading to dominant trap‐assisted recombination and resultant exciton formation on the sensitizer. This is further supported by the ideality factor (*η*) of > 2 (see the inset of Figure 1c).^[^ ^50^, ^51^ ^]^ Thus, given the fulfilment of the three requirements, we can attain efficient sensitizing process in our hyper‐OLED system (vide infra).

**Figure 2:**
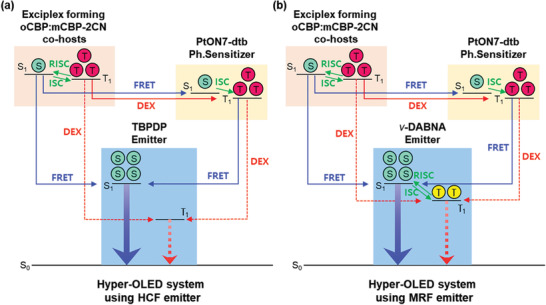
Schematic mechanisms for hyper‐OLED system using a) TBPDP and b) *ν*‐DABNA emitters. Here, we note that the working mechanism of hyper‐OLEDs is based on the use of exciplex‐forming co‐hosts and Ph‐sensitizer, and demonstrate excited singlet and triplet state, ground state, and Förster and Dexter energy transfer by S_1_, T_1_, S_0_, FRET, and DEX, respectively.

The operating principle of the hyper‐OLEDs using either TBPDP or *ν*‐DABNA is schematically described in **Figure** **2**. In the TBPDP hyper‐OLED (see Figure 2a), it is essential that the FRET from the sensitizer to TBPDP be efficient for harvesting triplet excitons for emission. On the other hand, the Dexter energy transfer, leading to non‐radiative loss of the triplets, should be suppressed by keeping the intermolecular spacing between the sensitizer and the emitter so that the triplets are not quenched in the latter. This is achieved by introducing a bulky group on the core of TBPDP and by using low doping concentration (<3%) of the emitters.

In the *ν*‐DABNA hyper‐OLED (see Figure 2b), even though the Dexter energy transfer occurs between the sensitizer and the emitter, the MRF *ν*‐DABNA, which also is a TADF material, can upconvert triplet into singlet excitons via the reverse intersystem crossing (RISC), owing to its small singlet–triplet energy gap (*Δ*E_ST_ ≈ 0.02 eV). Regardless, in terms of the device lifetime, it is of critical importance to minimize the Dexter energy transfer and the direct recombination on the MRF emitters owing to their long exciton decay time (*τ* ≈ 2.2 µs). In this regard, our MRF‐based hyper‐OLED was designed such that the excitons are directly formed on either the exciplex‐forming co‐host (oCBP:mCBP‐2CN) or the sensitizer rather than on the emitter, which was supported by the HOD and EOD characteristics and ideality factor (see requirement (iii) and Figure 1). Therefore, we can ensure the dominant FRET from the T_1_ state of the sensitizer to the S_1_ state of the emitters rather than the Dexter transfer and the effective prevention of the direct exciton formation by charge trapping on the fluorescent emitters (vide infra).

### 2.2 Photophysical Characteristics

**Figure 3:**
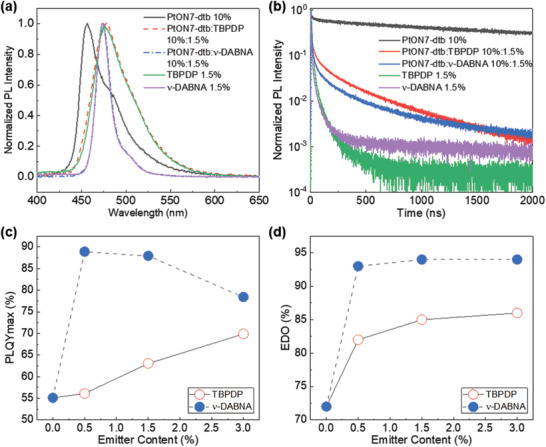
a) Normalized steady‐state and b) transient PL spectra of PtON7‐dtb 10%, PtON7‐dtb:TBPDP = 10%:1.5%, PtON7‐dtb:*ν*‐DABNA = 10%:1.5%, TBPDP 1.5%, and *ν*‐DABNA 1.5% in oCBP:mCBP‐2CN (5:5) matrix. c) PLQYmax and d) emitting dipole orientation (EDO) as a function of TBPDP and *ν*‐DABNA emitter content for PtON7‐dtb 10% in oCBP:mCBP‐2CN (5:5) matrix.

We investigated the photophysical characteristics of the device EMLs by measuring the steady‐state and transient PL (Figure S2, Supporting Information). The host in the EML films consists of oCBP and mCBP‐2CN at a 1:1 ratio and has PtON7‐dtp as a sensitizer doped at a 10 vol% concentration. Either TBPDP or *ν*‐DABNA was doped at a 1.5 vol% concentration as an emitter in the EML (each denoted as TBPDP EML and *ν*‐DABNA EML, respectively). In the steady‐state PL spectra in **Figure** **3**a, the phosphorescent EML (PtON7‐dtb only) exhibited the dominant peak of PL spectrum at *λ*_max_ = 457 nm (phosphorescence with T_1_ = 2.71 eV) with a FWHM of ≈46 nm. A broad PL spectrum was observed for the TBPDP EML (*λ*_max_ = 478 nm, S_1_ = 2.59 eV, and FWHM ≈51 nm), whereas the *ν*‐DABNA EML exhibited narrow PL spectrum (*λ*_max_ = 473 nm, S_1_ = 2.62 eV, and FWHM ≈20 nm). Interestingly, PL spectra of both hyper‐OLED EMLs are almost identical to those of the TBPDP and *ν*‐DABNA fluorescence, indicating that the PtON7‐dpt acts only as the sensitizer not as the emitter. As shown in Figure 3b, an efficient FRET from the sensitizer to the fluorophores can be inferred from reduced exciton decay time for the TBPDP (*τ* = 0.39 µs) or *ν*‐DABNA EMLs (*τ* = 0.84 µs), compared to that of the phosphorescent EML of *τ* = 3.3 µs. Here, the exciton decay time (*τ*) is an intensity‐weighted average of those extracted by fitting the transient PL with a multi‐exponential function. As depicted in Figure 3c, the PLQYs of the TBPDP (63.0%) and *ν*‐DABNA EMLs (87.9%) are significantly higher than that of the phosphorescent EML (55.1%), irrespective of the concentration of the TBPDP and *ν*‐DABNA emitters. As a result, the radiative decay rate (*k*_r_) was significantly increased from *k*_r_ = 1.7 × 10^5^ s^−1^ for the phosphorescent EML to 16.3 × 10^5^ s^−1^ and 10.5 × 10^5^ s^−1^ for TBPDP and *ν*‐DABNA EMLs, respectively. It is noteworthy that increase in PLQY and reduction in exciton lifetime, resulting in faster radiative decay rate, can be attributed to the efficient energy transfer from host and sensitizer to emitters based on the large spectral overlap between emission of host and sensitizer and absorption of emitter (see Figure 1b; and Figure S3, Supporting Information). In addition, as shown in Figure 3d, the emitting dipole orientations (EDOs) of the TBPDP and *ν*‐DABNA EMLs were noticeably increased, reaching up to 86% and 94%, respectively. Note that these values are close to those of the TBPDP (87%) and *ν*‐DABNA fluorescent EMLs (95%) (see Figures S4 and S5, Supporting Information). As a result, the improvement in the light outcoupling efficiency and PLQY of the TBPDP and *ν*‐DABNA EMLs contribute to enhanced EQEs of the hyper‐OLEDs based on such EMLs, compared to the conventional phosphorescent OLED.

### 2.3 Effect of TBPDP and *ν*‐DABNA Concentration on the OLED Performance

**Figure 4:**
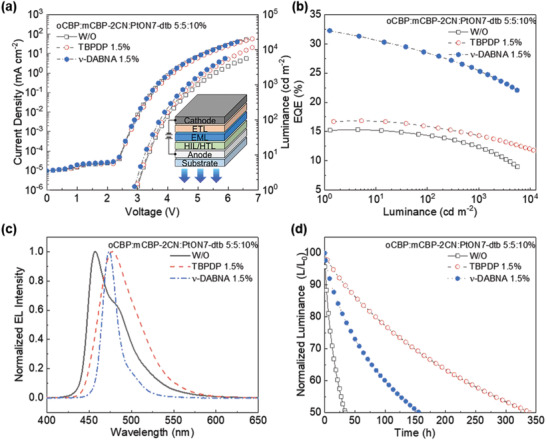
a) EQE–L curves, and b) normalized electroluminescence (EL) spectra of bottom‐emission for the control device (PtON7‐dtb 10%) and 1.5% TBPDP and *ν*‐DABNA hyper‐OLED devices at luminance of 1000 cd m^−2^. c) Normalized luminance of the control device (PtON7‐dtb 10%) and d) 1.5% TBPDP and *ν*‐DABNA hyper‐OLED devices as a function of the operating time at a constant current, corresponding to initial L = 1000 cd m^−2^.

We investigated the effect of the TBPDP and *ν*‐DABNA concentration (0–3 vol%) on the OLED performance (see Figure S6, Supporting Information). The key parameters are summarized in **Table** **1**. As shown in **Figure** **4**A, there is only a negligible shift in the *J–V* curves of the devices regardless of TBPDP and *ν*‐DABNA. This indicates that charge trapping less likely occurs on TBPDP or *ν*‐DABNA in the EML, which is in good agreement with the HOD and EOD characteristics. As shown in **Figure** **4**b; and Figure S6, Supporting Information, both TBPDP and *ν*‐DABNA hyper‐OLEDs show increased EQE_max_ = 16.9% and 32.2%, respectively, compared to the phosphorescent OLED (15.3%) and 1.5% emitter‐only OLEDs (7.5% and 30.5% for TBPDP‐only and *ν*‐DABNA‐only OLEDs, respectively). In particular, the *ν*‐DABNA hyper‐OLED exhibits EQE of 25.4% at 1000 cd m^−2^, which is significantly higher than that of the phosphorescent OLED (12.4%) and the TBPDP hyper‐OLED (14.3%). The increase in the EQE of hyper‐OLEDs is attributed to the increased PLQY and outcoupling efficiency (see Figure S7, Supporting Information). Interestingly, it is worth mentioning that EQE roll‐off for both TBPDP (15.1%) and *ν*‐DABNA (21.2%) hyper‐OLEDs at 1000 cd m^−2^ is obviously suppressed, compared to 1.5% TBPDP‐only (33.5%) and 1.5% *ν*‐DABNA ‐only OLEDs (29.2%) at 1000 cd m^−2^. The reduced roll‐off can be attributed to the relatively short exciton lifetime and less generated triplet exciton on the emittters. In other words, the excitons effectively radiative decay in TBPDP and *ν*‐DABNA EMLs and triplet exciton population is quickly depleted and less accumulated so that the triplet‐related annihilation is less likely to happen.

**Table 1:** Photophysical characteristics and OLED performance parameters of bottom‐emission control and hyper‐OLED devices

						CE [cd A^−1^]		EQE [%]					
PtON7‐dtb[%]	TBPDP[%]	*ν*‐DABNA[%]	PLQY_max_[%]	tau[µs]	EDO[%]	max	1000 cd m^−2^	max	1000 cd m^−2^	CIE	*λ*_max_[nm]	FWHM [nm]	T50[h]
10	—	—	55.1	3.30	72	18.7	15.2	15.3	12.4	(0.139, 0.153)	457	46	34.4
10	0.5	—	56.1	1.07	82	27.3	22.9	17.9	14.9	(0.131, 0.223)	474	56	143.3
10	1.5	—	63.0	0.39	85	28.9	24.6	16.9	14.3	(0.128, 0.271)	478	51	339.2
10	3	—	69.9	0.16	86	27.9	22.7	15.0	12.2	(0.129, 0.309)	480	51	452.8
0	1.5	—	74.3	0.03	87	11.6	7.5	6.6	4.4	(0.126, 0.270)	476	51	92.9
10	—	0.5	88.9	1.35	93	25.3	17.7	24.9	16.5	(0.128, 0.138)	471	29	68.4
10	—	1.5	87.9	0.84	94	32.0	25.1	32.2	25.4	(0.111, 0.141)	473	20	156.3
10	—	3	78.4	0.69	94	31.7	23.9	30.7	23.2	(0.107, 0.154)	475	20	116.8
0	—	1.5	83.7	2.20	95	30.1	21.1	30.5	21.6	(0.109, 0.141)	474	20	77.9

**Table 2:** Performance parameters of top‐emission control and hyper‐OLED devices

			CE [cd A^−1^]					
PtON7‐dtb [%]	TBPDP [%]	*ν*‐DABNA [%]	max	1000 cd m^−2^	CIE	*λ*_max_ [nm]	FWHM [nm]	T50 [h]
10	—	—	13.7	8.49	(0.134, 0.060)	462	25	13.4
10	1.5	—	23.8	16.11	(0.109, 0.134)	476	25	192.2
10	—	1.5	48.9	37.81	(0.115, 0.091)	473	17	253.8

The electroluminescence (EL) spectra of the TBPDP and *ν*‐DABNA hyper‐OLEDs are almost identical to those of the conventional TBPDP‐ and *ν*‐DABNA fluorescent OLEDs, which is in good agreement with the PL spectra (see Figure 4c; and Figure S6, Supporting Information). The FWHM of the EL spectrum of the *ν*‐DABNA hyper‐OLED is ≈20 nm, whereas that of the phosphorescent OLED and the TBPDP hyper‐OLED are ≈46 and 51 nm, respectively. The narrow FWHM of the *ν*‐DABNA hyper‐OLED results in both high efficiency and long device lifetime in a top‐emission structure owing to the strengthened micro‐cavity effect (vide infra). As shown in Figure 4d, an operational lifetime of the hyper‐OLEDs was noticeably increased; T50 for v‐DABDA and TBPDP hyper‐OLEDs = 156.3 and 339.2 h, respectively, compared to that of the phosphorescent OLED of T50 = 77.9h.

We investigated the photochemical stability for the EMLs with and without TBPDP and *ν*‐DABNA by tracking the change of their PL intensity before and after UV‐laser exposure at the excitation wavelength of 325 nm for 180 min (Figure S7, Supporting Information). Interestingly, the PL intensity reduction for the TBPDP‐ and *ν*‐DABNA EMLs (−10.6% and −15.5%, respectively) is less severe than the phosphorescent EML (−24.6%). This indicates that the TBPDP‐ and *ν*‐DABNA EMLs enhances photochemical stability as the exciton residence time is decreased by the FRET of triplet excitons to the emitters. Thus, the suppressed EQE roll‐off and prolonged device lifetime are mainly attributed to the prevention of the direct recombination on the emitters and the reduced exciton decay time via the efficient FRET from the sensitizer to emitter, leading to the reduced triplet‐related annihilations in the hyper‐OLEDs.^[^ ^27^, ^52^ ^]^ This is in good agreement with the reduced triplet exciton density on both emitters based on the device simulation result, as will be discussed below.

### 2.4 Device Simulation Based on the Kinetic Monte Carlo Method

**Figure 5:**
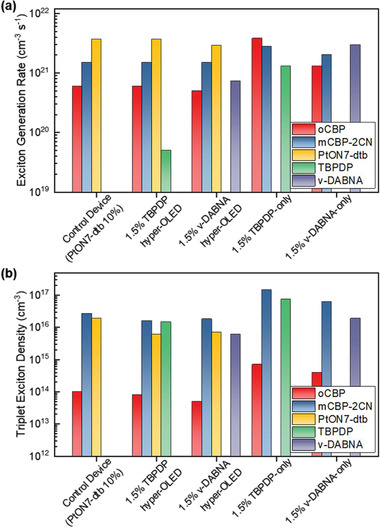
a) Simulated exciton generation rate and b) triplet exciton density at host, sensitizer, and emitter for control device, 1.5% TBPDP and 1.5% *ν*‐DABNA hyper‐OLED devices, and 1.5% TBPDP‐only and 1.5% *ν*‐DABNA‐only devices

In order to verify an operating principle of our hyper‐OLEDs, we performed a multilayer‐device simulation based on the kinetic Monte Carlo method. The simulation is based on the probabilistic transfer of charges and excitons according to pre‐determined rate parameters. The details of the device modeling and simulation are provided in the Supporting Information. The simulation parameters are summarized in Tables S3 and S4. In the phosphorescent and TBPDP hyper‐OLEDs, a dominant portion of the holes (99.5%) resides in the PtON7‐dtb molecules, as the HOMO energy level for PtON7‐dtb is shallower than that for the host (oCBP) and the fluorophore (TBPDP) by 0.60 and 0.28 eV, respectively. In contrast, most of the electrons reside in the host matrix (oCBP:mCBP‐2CN), because the host molecules comprise most of the EML, and the lowest unoccupied molecular orbital (LUMO) energy levels of the constituent molecules are similar except for *ν*‐DABNA. Thus, the ratio of electron densities does not vary significantly in the other devices considered in this study. The recombination of a hole and an electron occurs at a rate dependent on the densities and mobilities of the charge carriers. As a result, excitons are created at the rate of 3.7 × 10^21^ and 2.1 × 10^21^ cm^−3^s^−1^ in PtON7‐dtb and the host in the phosphorescent OLED, respectively (see **Figure** **5**a). In the TBPDP hyper‐OLED, TBPDP does not hold carriers significantly and only 0.8% of the excitons are created in the TBPDP emitter. Although the formation of excitons in the phosphorescent OLED does not differ significantly from that in the TBPDP hyper‐OLED, the densities of triplet excitons residing in PtON7‐dtb during the device operation are very different. The triplet exciton density on PtON7‐dtb is found to be 0.6 × 10^16^ cm^−3^ in the 1.5% TBPDP hyper‐OLED, which is 68% lower than that in the control device in Figure 5b. This is attributed to the efficient energy transfer from PtON7‐dtb to TBPDP in the hyper‐OLED. Moreover, the density of triplet excitons residing at TBPDP in the hyper‐OLED is much smaller than that in the TBPDP‐based fluorescent device (1.5 vs 7.4 in units of 10^16^ cm^−3^), because the direct formation of excitons in TBPDP is successfully reduced in the presence of PtON7‐dtb. In the *ν*‐DABNA hyper‐OLED, most of the excitons are created in PtON7‐dtb and the host as in the TBPDP hyper‐OLED. However, a large number of excitons is created at the rate of 0.73 × 10^21^ cm^−3^s^−1^ in *ν*‐DABNA, because approximately one‐fifth of the holes are trapped in *ν*‐DABNA, which has the shallowest HOMO energy. Nevertheless, it is less than the number of excitons created in *ν*‐DABNA in the *ν*‐DABNA fluorescent OLED (0.73 vs 2.96 in units of 10^21^ cm^−3^ s^−1^). In the same manner as in the TBPDP hyper‐OLED, the number of triplet excitons in PtON7‐dtb in the *ν*‐DABNA hyper‐OLED is reduced by 63% when compared with that in the control device. Efficient elimination of triplet excitons from PtON7‐dtb and the fluorescent emitters is important for improving the stability of the device.

### 2.5 Top‐Emission OLED Performance

**Figure 6:**
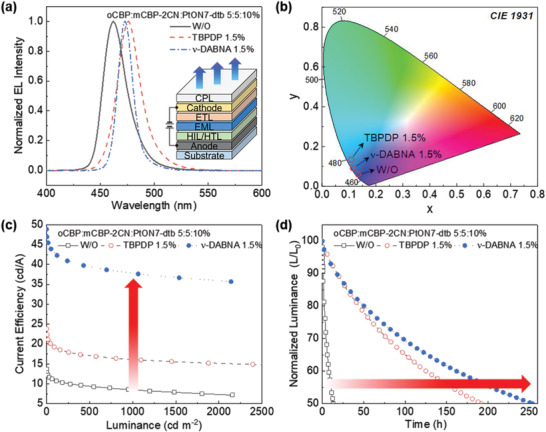
a) Normalized EL spectra of top‐emission control device and 1.5% TBPDP and 1.5% *ν*‐DABNA hyper‐OLED devices at luminance of 1000 cd m^−2^, corresponding to b) Commission Internationale d'Eclairage (CIE 1931) chromaticity coordinates. c) CE‐L characteristics and d) normalized luminance of top‐emission control device and 1.5% TBPDP and 1.5% *ν*‐DABNA hyper‐OLED devices as a function of operating time at a constant current, corresponding to initial L = 1000 cd m^−2^.

To further demonstrate the feasibility of our proposed device for active‐matrix display applications, we fabricated top‐emission hyper‐OLEDs (see details in the Supporting Information). The key parameters for top‐emission OLEDs are summarized in **Table** **2**. As shown in **Figure** **6**a, the top‐emission OLEDs exhibit narrower FWHM than the bottom‐emission OLEDs. As a result, deep blue emission was obtained with the CIE chromaticity coordinates of (0.134, 0.060), (0.109, 0.134), and (0.115, 0.091) for the control (i.e., phosphorescent OLED), TBPDP and *ν*‐DABNA hyper‐OLEDs, respectively (see Figure 6b). A remarkable enhancement is seen in current efficiency (CE) for the *ν*‐DABNA hyper‐OLED (CEmax = 48.9 cd A^−1^, CE at 1000 cd m^−2^ = 37.8 cd A^−1^) owing to the micro‐cavity effect that eliminates waveguide loss and a narrow spectrum of *ν*‐DABNA (see Figure 6c). The CE is much higher than that of the control device (CEmax = 13.7 cd A^−1^, CE at 1000 cd m^−2^ = 8.5 cd A^−1^) and the TBPDP hyper‐OLED (CEmax = 23.8 cd A^−1^, CE at 1000 cd m^−2^ = 16.1 cd A^−1^). As shown in Figure 6d, the operational lifetime (T50) at 1000 cd m^−2^ for the *ν*‐DABNA hyper‐OLED is significantly longer by 18.9 times from 13.4 h (control device) to 253.8 h, which is even higher than that of the TBPDP hyper‐OLED (≈192.2 h). To the best of our knowledge, *ν*‐DABNA hyper‐OLED shows one of the best device performances for deep‐blue OLEDs with CIEy below 0.3 (see Table S5, Supporting Information). In particular, superior device performance for top‐emission *ν*‐DABNA hyper‐OLED is achieved in terms of the driving voltage (≈4.5 V at 1000 cd m^−2^), current efficiency (≈48.9 cd A^−1^), and device lifetime (≈253.8 h), as compared to the best reported values to date for PPCzTRz:*ν*‐DABNA and PCzTRz:*ν*‐DABNA triplet‐exciton‐distributed TADF devices.^[^ ^53^ ^]^

## 3 Conclusion

We demonstrated highly efficient and stable hyper‐OLEDs using PtON7‐dtb as the sensitizer and either TBPDP or *ν*‐DABNA as the luminescent fluorophore. The proposed, hyper‐OLEDs exhibit less charge trapping on the fluorescent emitters and efficient FRET from the T_1_ state of the PtON7‐dtb sensitizer to the S_1_ states of the TBPDP and *ν*‐DABNA emitters, resulting in improved device efficiency and extended device lifetime owing to the increased PLQY/EDO and reduced exciton lifetime. As a result, the top‐emission hyper‐OLED using *ν*‐DABNA attained deep blue emission with a CIE coordinate of (0.115, 0.091) as well as significant increase in the maximum CE (48.9 cd A^−1^) and 19‐fold improved device lifetime reaching up to T50 = 253.8 h at 1000 cd m^−2^. Further improvement of the long‐lasting, high‐efficiency deep‐blue hyper OLEDs in future will realize their successful adoption in the practical applications.

## 4 Experimental Section

### Materials

All chemicals were purchased from commercial suppliers and used without further purification, or were synthesized as previously reported.

### Solution and Thin Film Characterization

UV–visible absorption and photoluminescence (PL) spectra of the solution in toluene (10^−5^ m) were conducted using a UV–vis–NIR spectrophotometer (Cary 5000, Agilent Technologies) and fluorescence spectrophotometer (F‐7000, Hitachi). PL spectrum and photoluminescence quantum yield (PLQY) measurement were performed using a PLQY spectrometer (Quantaurus‐QY, C11347, Hamamatsu Photonics). Angle‐dependent PL was examined using commercial equipment (OLED‐PLA, Luxol). Transient photoluminescence was measured using a fluorescence lifetime spectrometer (FluoTime 300, PicoQuant). The thin films for optical characterization were fabricated using the same device fabrication process.

### Device Fabrication and Characterization

Pre‐patterned 50‐nm‐thick ITO‐glass substrates were treated with a wet‐cleaning process (acetone, isopropyl alcohol, and deionized water), followed by a dry‐cleaning process (UV‐ozone treatment). The organic layers were consecutively deposited on ITO‐glass substrates in thermal evaporator chambers at the rate of 0.03 to 1 Å s^−1^ under high vacuum (<1.0 × 10^−6^ torr) (note that the emissive layer was co‐deposited at the rate of 0.001 to 0.5 Å s^−1^). 150‐nm‐thick Al electrodes were thermally evaporated at the rate of 1.5 Å s^−1^ via shadow masks. The device area was 4 mm^2^, defined by an overlap between the anode and cathode electrodes. All devices were encapsulated in a nitrogen‐filled glovebox using UV‐curable resin with glass capsule lids prior to device characterization. The current density–voltage–luminance (*J–V–L*) characteristics were measured using a source meter (2636B, Keithley) and radiospectrometer (SR‐3AR, Topcon). The device lifetime was recorded from an initial luminance of 1000 cd m^−2^ at room temperature by using an OLED lifetime tester (M6000 PMX, McScience). The photochemical stability was examined using a He–Cd laser (excitation wavelength = 325 nm, 3.5 mW, IK3202R‐D, KIMMON KOHA) with a fluorescent spectrometer (QE65 Pro, Ocean Optics).

## Conflict of Interest

The authors declare no conflict of interest.

## Supporting information



Supporting Information

## Data Availability

Research data are not shared.
