# Supplementary material for: Improved Efficiency and Lifetime of Deep‐Blue Hyperfluorescent Organic Light‐Emitting Diode using Pt(II) Complex as Phosphorescent Sensitizer
Source: Adv Sci (Weinh). 2021 Jun 16;8(16):2100586. doi: 10.1002/advs.202100586 (PMC8373157; doi:10.1002/advs.202100586)
Supplement: Supplementary file 1 — Supporting Information [file ADVS-8-2100586-s001.pdf]

© 2021 Wiley-VCH GmbH

ADVANCED SCIENCE
Open Access

Supporting Information

for *Adv. Sci.,* DOI: 10.1002/advs.202100586

Improved efficiency and lifetime of deep-blue hyperfluorescent organic light-emitting diode using Pt(II) complex as phosphorescent sensitizer

Sungho Nam, Ji Whan Kim, Hye Jin Bae, Yusuke Makida Maruyama, Daun Jeong, Joonghyuk Kim, Jong Soo Kim, Won-Joon Son, Hyein Jeong, Jaesang Lee, Soo-Ghang Ihn, Hyeonho Choi**

Supporting Information

Improved efficiency and lifetime of deep-blue hyperfluorescent organic light-emitting diode using Pt(II) complex as phosphorescent sensitizer

Sungho Nam, Ji Whan Kim, Hye Jin Bae, Yusuke Makida Maruyama, Daun Jeong, Joonghyuk Kim, Jong Soo Kim, Won-Joon Son, Hyein Jeong, Jaesang Lee, Soo-Ghang Ihn, Hyeonho Choi**

Dr. S. Nam, Dr. J. W. Kim, Dr. H. J. Bae, Dr. Y. M. Maruyama, Dr. J. Kim, Dr. J. S. Kim, Dr. S.-G. Ihn, Dr. H. Choi
Samsung Advanced Institute of Technology, Samsung Electronics Co., Ltd., 130 Samsung-ro, Suwon-si, Gyeonggi-do 16678, Republic of Korea
E-mail: sg.ihn@samsung.com, hono.choi@samsung.com

Dr. D. Jeong, Dr. W-J. Son
Data and Information Technology Center, Samsung Electronics Co., Ltd., 1 Samsungjeonja-ro, Hwaseong-si, Gyeonggi-do 18448, Republic of Korea

Dr. H. Jeong
Display Research Center, Samsung Display Co., 1 Samsung-ro, Yongin-si, Gyeonggi-do, 17113, Republic of Korea

Prof. J. Lee
Department of Electrical and Computer Engineering, Inter-University Semiconductor Research Center, Seoul National University, Seoul 08826, Republic of Korea

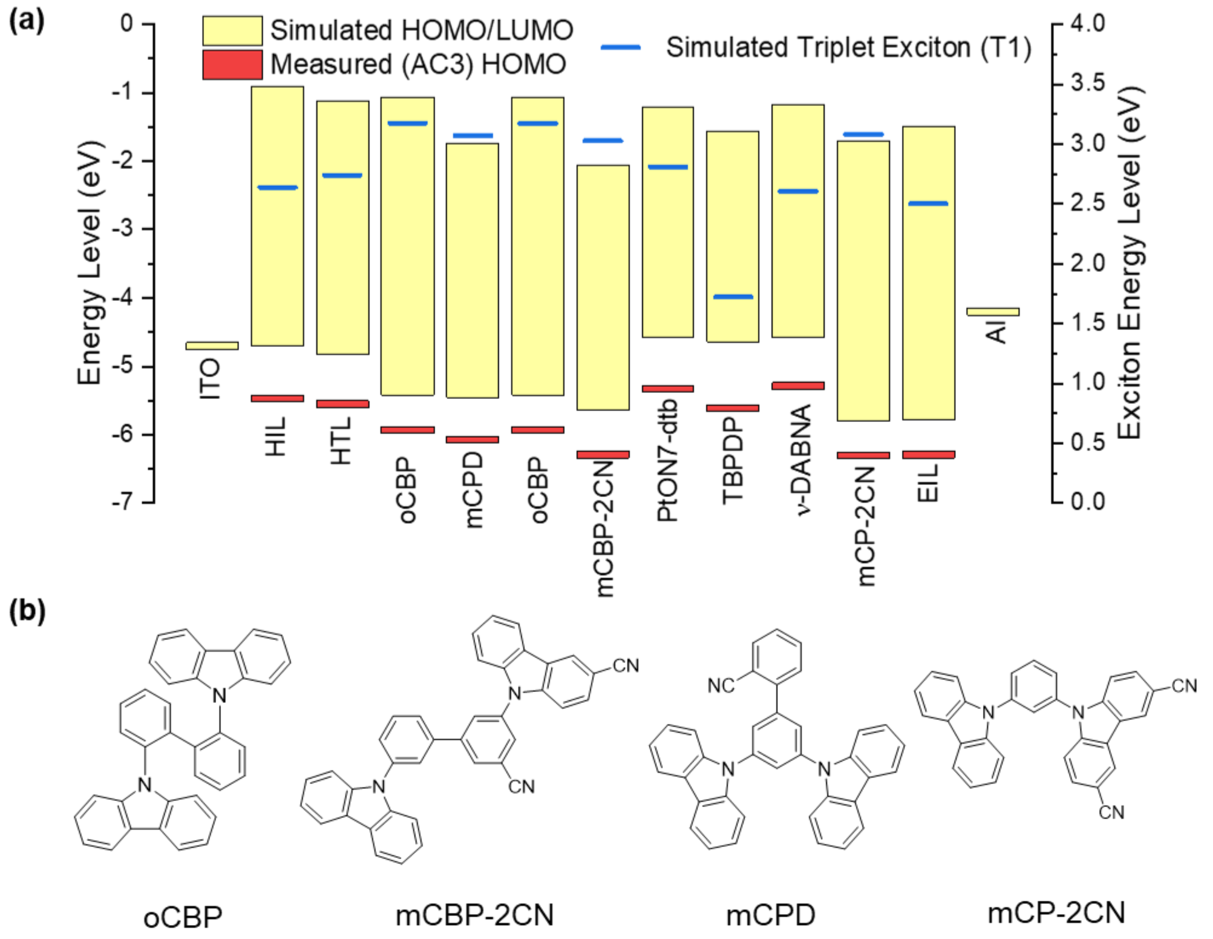

Figure S1. (a) Highest occupied molecular orbital (HOMO), lowest unoccupied molecular orbital (LUMO), and triplet energy levels used in this work, calculated from density functional theory (DFT) with 6-31G(d,p) basis set and HOMO energy level measured by AC3. (b) Chemical structures of oCBP, mCBP-2CN, mCPD, and mCP-2CN.

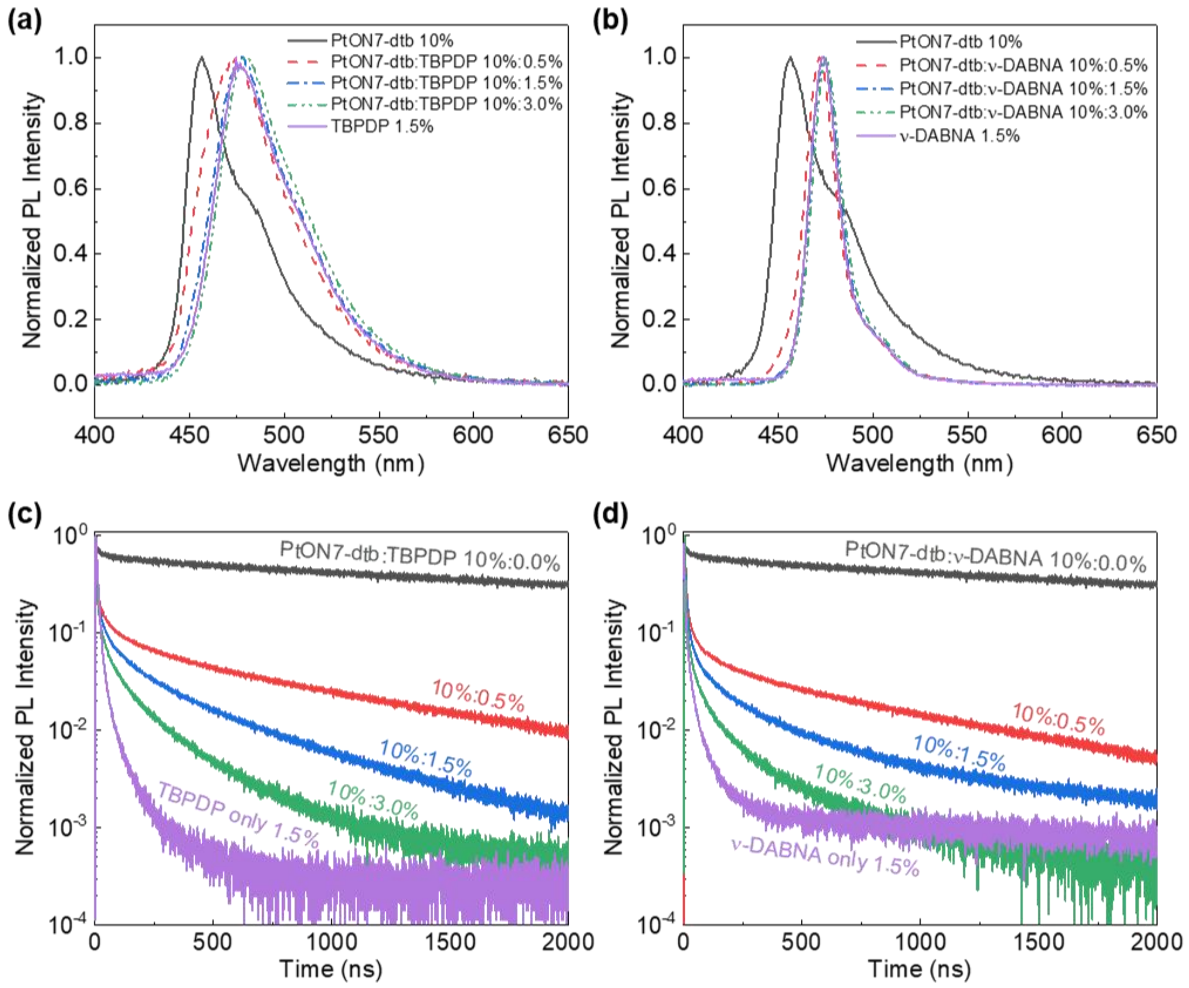

Figure S2. (a,b) Normalized steady-state and (c,d) transient PL spectra of PtON7-dtb 10% in oCBP:mCBP-2CN (5:5) matrix according to the TBPDP and ν-DABNA content.

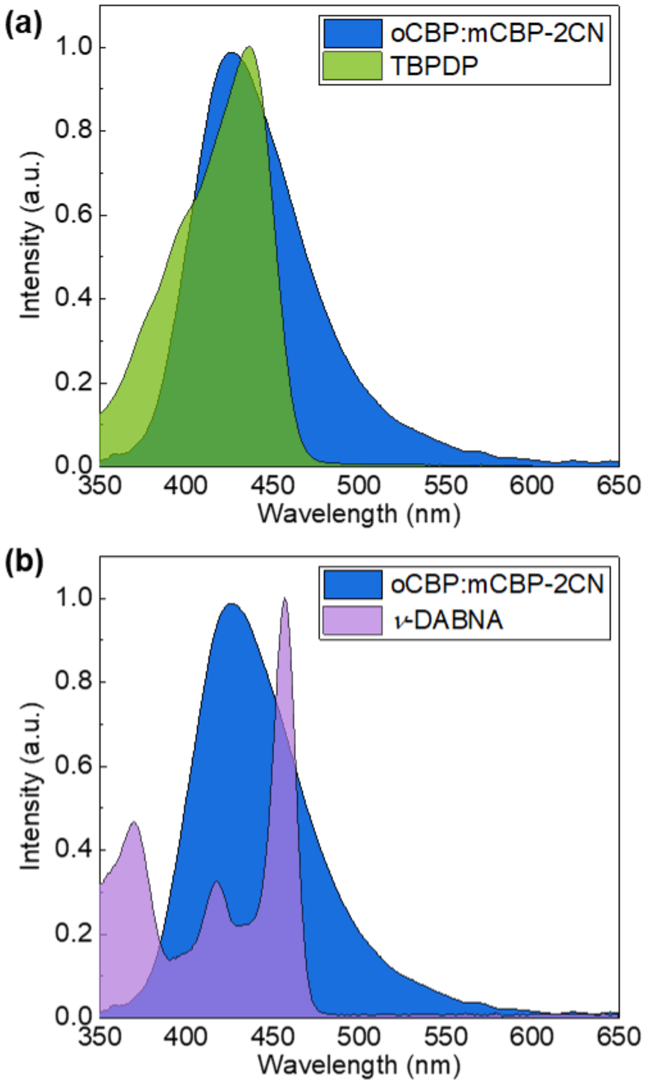

Figure S3. Spectral overlap region between oCBP:mCBP-2CN (5:5) photoluminescence in solid-state film and absorption spectra of (a) TBPDP and (b) ν-DABNA emitters in toluene (10^{-5} M solution).

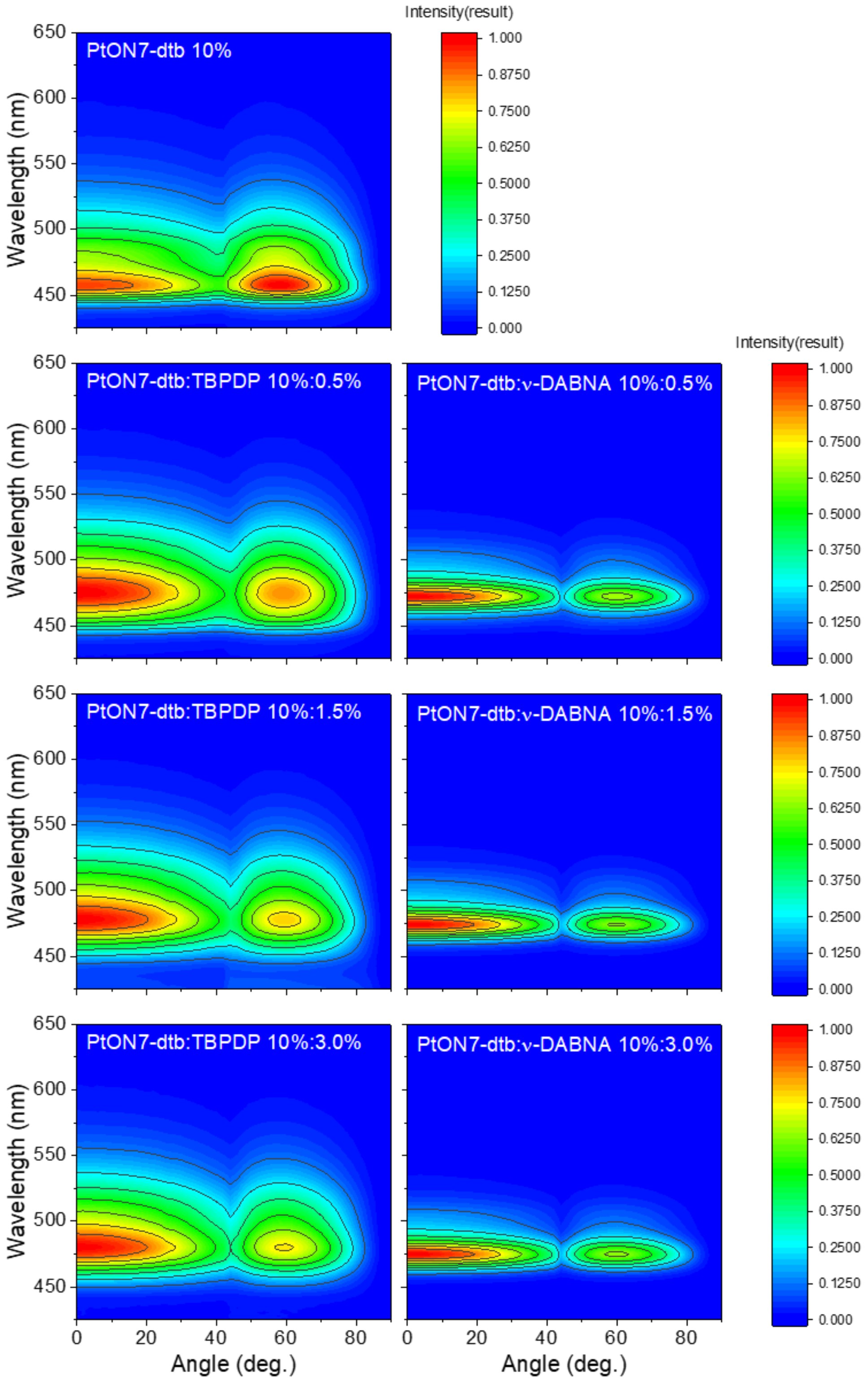

Figure S4. Angle-dependent PL spectra of PtON7-dtb 10% in oCBP:mCBP-2CN (5:5) matrix according to the TBPDP and ν-DABNA content.

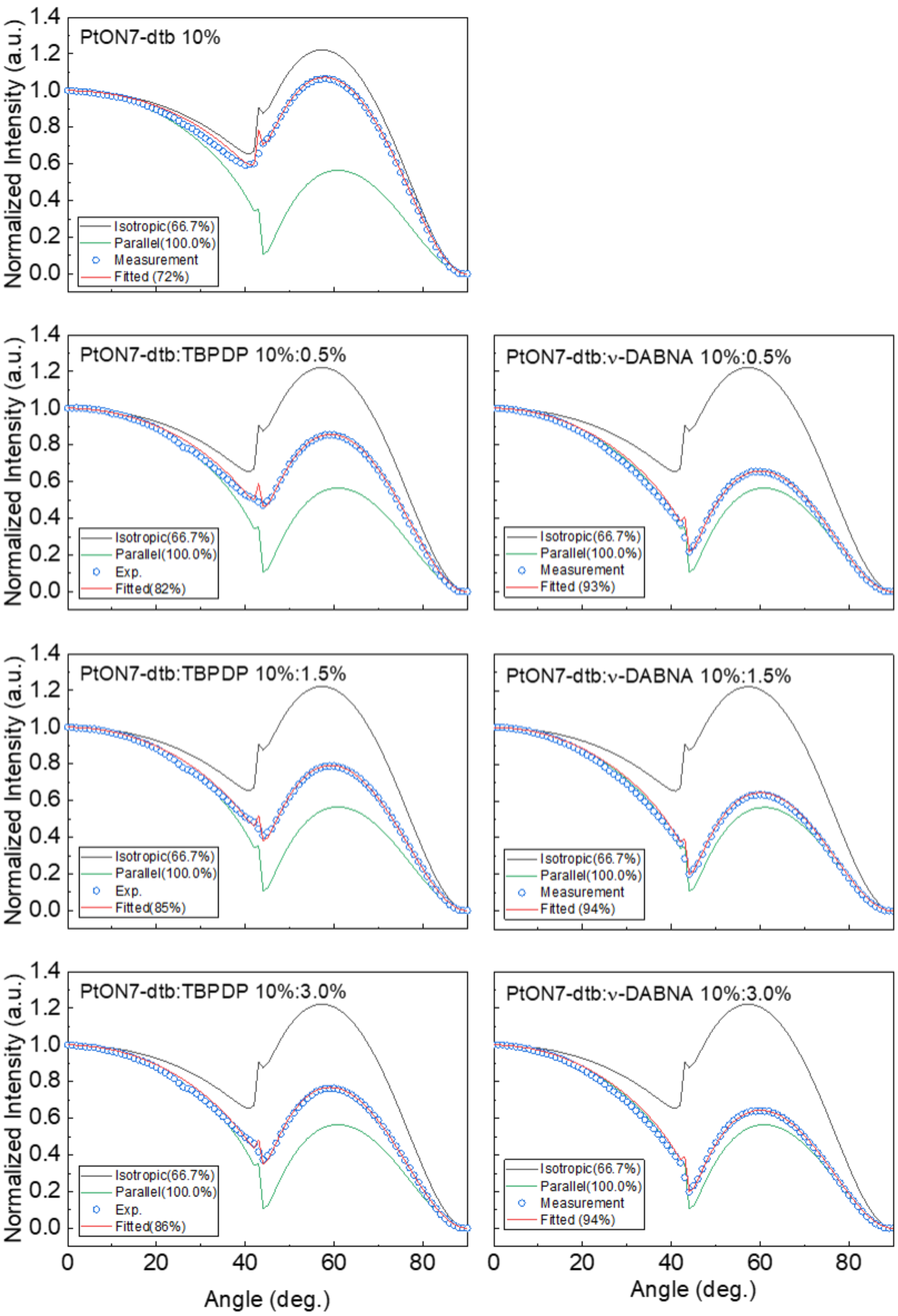

Figure S5. Angular p-polarized emission profiles of PtON7-dtb 10% in oCBP:mCBP-2CN (5:5) matrix according to the TBPDP and ν-DABNA content.

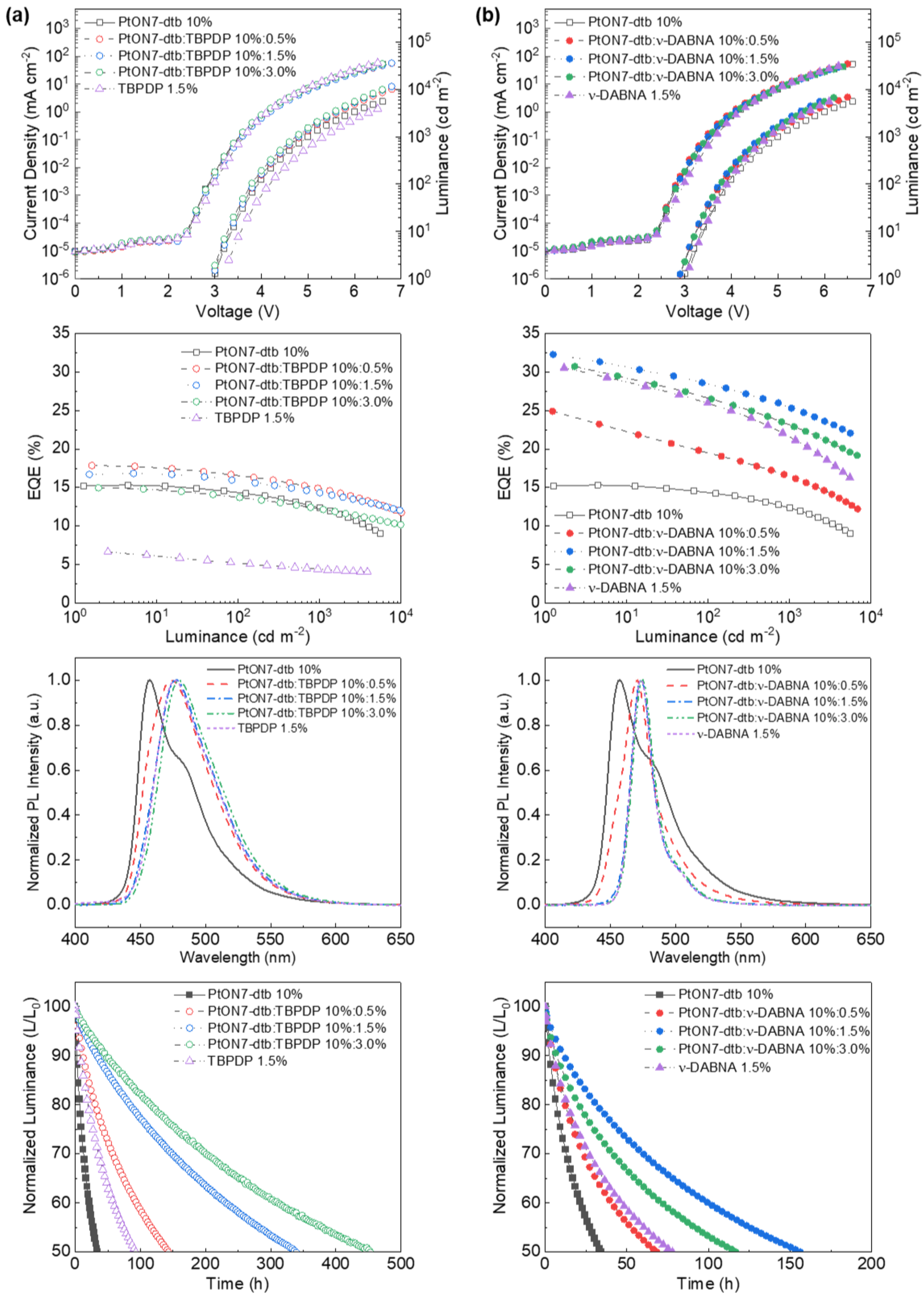

Figure S6. J–V–L and EQE–L curves, normalized EL spectra of bottom-emission hyper-OLEDs at L of 1000 cd m^{-2}, and normalized luminance of hyper-OLEDs over the operating time at a constant current, corresponding to initial L = 1000 cd m^{-2}: (a) TBPDP and (b) ν-DABNA emitters.

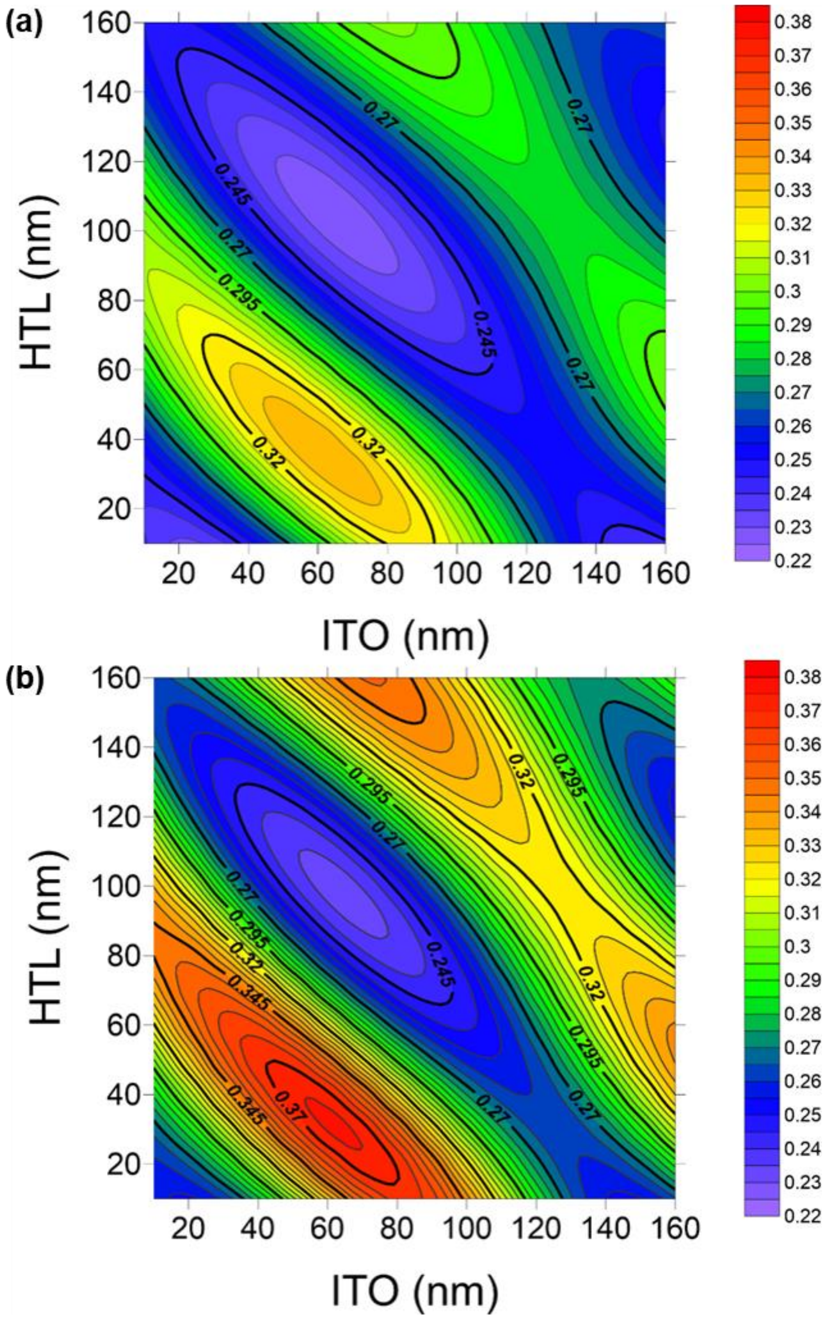

Figure S7. Calculated maximum EQE as a function of ITO and HTL thickness (here note that we reflect the simulation parameters such as refractive index, emission position, and light outcoupling and assume PLQY of 100%): Hyper-OLEDs using (A) TBPDP and (B) ν-DABNA emitters.

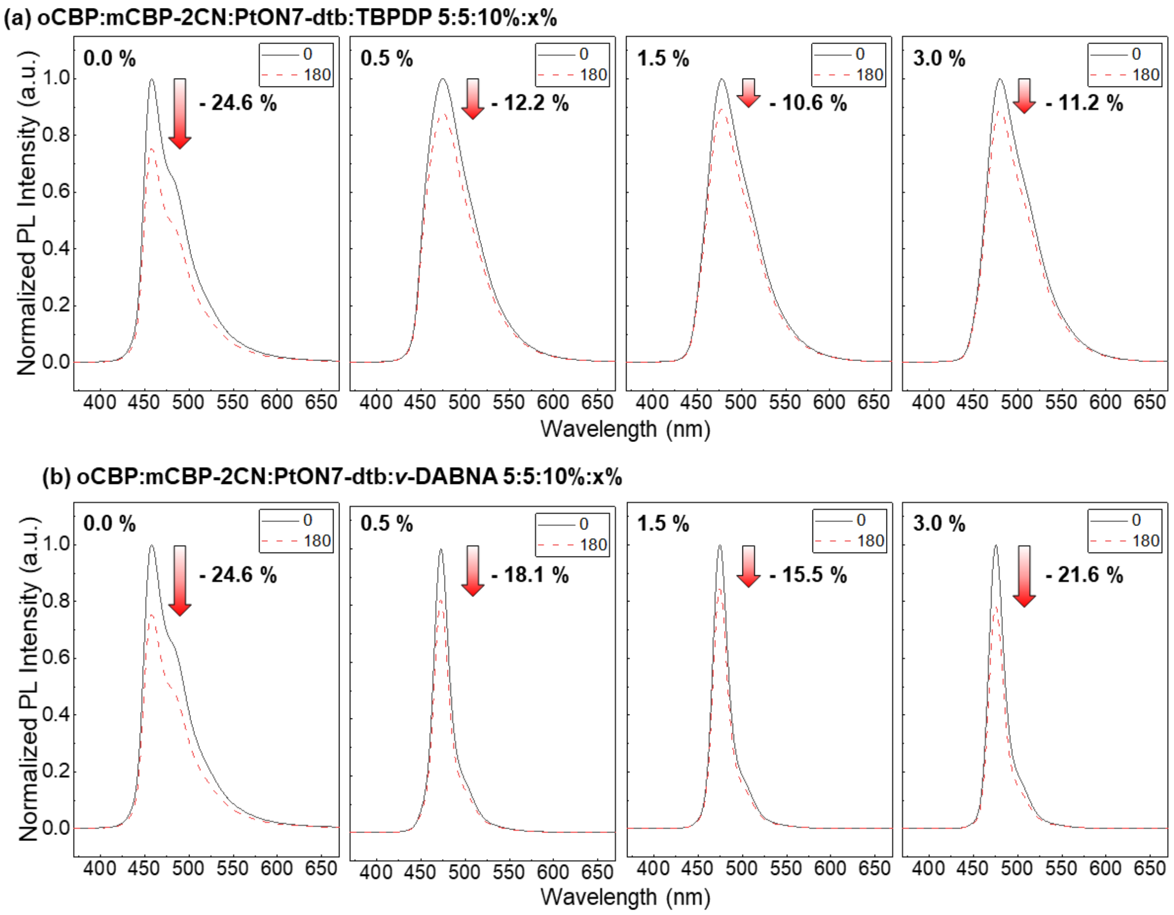

Figure S8. PL spectra upon UV exposure at excitation wavelength = 325 nm before and after 180 min: (a) PtON7-dtb:TBPDP 10%:x% and (b) PtON7-dtb:ν-DABNA 10%:x% in oCBP:mCBP-2CN (5:5) matrix.

Details of bottom-emission OLED device structures: The optimized bottom-emission OLED device structure was designed as follows: ITO (50 nm) / hole injection layer (HIL, 10 nm, p-doped (3 wt%, NDP series, purchased from Novaled AG) N-([1,10-biphenyl]-4-yl)-9,9-dimethyl-N-(4-(9-phenyl-9Hcarbazol-3-yl) phenyl)-9H-fluoren-2-amine (BCFA)) / hole transporting layer (HTL, 125 nm, BCFA) / HTL 2 (10 nm, HT series, supplied from Samsung SDI) / exciton blocking layer (5 nm/5 nm, oCBP/mCPD) / EML (40 nm, oCBP:mCBP-2CN:PtON7-dtb:TBPDP or ν-DABNA 5:5:10%:x%) / hole blocking layer (HBL, 10 nm, mCP-2CN) / electron transport layer (ETL, 31 nm, 5:5, co-deposited NET series:NDN series, purchased from Novaled AG) / Al (100 nm).

We used the cascade double EBL (oCBP/mCPD) for efficient hole injection and effective exciton confinement in the EML and electron overflow into the HTL. In addition, we introduced the exciplex forming co-host of oCBP and mCBP-2CN with high triplet energy (>2.7 eV) and adopted mCP-2CN as the HBL to prevent hole leakage into the ET and confine the exciton within the EML.

Details of top-emission OLED device structures: The optimized top-emission OLED device structure was designed as follows: ITO/Ag alloy/ITO (150 nm) / hole injection layer (HIL, 10 nm, p-doped (3wt%, NDP series, purchased from Novaled AG) N-([1,10-biphenyl]-4-yl)-9,9-dimethyl-N-(4-(9-phenyl-9Hcarbazol-3-yl) phenyl)-9H-fluoren-2-amine (BCFA))[1,2] / hole transporting layer (HTL, 90 nm (control) and 95 nm (hyper-OLEDs), BCFA) / HTL 2 (10 nm, HT series, supplied from SDI) / exciton blocking layer (5 nm/5 nm, oCBP/mCPD) / EML (40 nm, oCBP:mCBP-2CN:PtON7-dtb:TBPDP or ν-DABNA 5:5:10%:1.5%) / hole blocking layer (HBL, 10 nm, mCP-2CN) / electron transporting layer (ETL, 31 nm, 5:5, co-deposited NET series:NDN series, purchased from Novaled AG) / Yb (1.5 nm) / AgMg (13 nm) / capping layer (CPL, 70 nm).

Modeling and simulation methods

We performed kinetic Monte Carlo (KMC) simulations to examine the charge transport and excitonic processes in the hyperfluorescent OLED device. The excitonic processes include the excitation of molecules, intersystem crossing, energy transfer, quenching, and (non)radiative decay. Excitons undergo quenching when the energy is transferred from an excited molecule to another excited molecule or a charged molecule, followed by a fast decay to its original state. It is assumed that all charge transfer and excitonic processes are independent discrete events in the dynamics in KMC simulations.[1] We monitored the state of all molecules in the device at each instance and obtained the profiles of charges, excitation events, and photon emission according to the distance from the anodes. We performed KMC simulations using LightForge software implemented in Nanomatch.[2] The KMC simulations require predefined rates of all microscopic processes. Next, we describe the setting of rates in our study.

In a weakly coupled regime, the Marcus theory is used for the calculation of charge (or energy) transfer rate denoted by k_{if} and expressed as

$$k_{\mathrm{if}} = \frac{2\pi}{\hbar} |J_{\mathrm{if}}|^2 \frac{1}{\sqrt{4\pi\lambda_{\mathrm{if}} k_{\mathrm{B}} T}} \exp\left(-\frac{(\lambda_{\mathrm{if}} + \Delta G_{\mathrm{if}})^2}{4\lambda_{\mathrm{if}} k_{\mathrm{B}} T}\right),$$

where J_{if} is the electronic coupling, λ_{if} is the reorganization energy, and ΔG_{if} is the difference in the Gibbs free energy of the initial and final states.[3] To calculate the Marcus rate, we need these microscopic parameters, which should be calculated quantum mechanically.

We consider the state energy as the Gibbs free energy assuming the entropy does not change significantly during the charge (or energy) transfer. The state energy strongly depends not only on the variation in the conformation of organic molecules but also on the local environment, which varies according to the material used.[4,5] The statistical mean of the state energy and the variance of the energy difference are key parameters in our device modeling in order to determine all the site energies, which involves charte and energy transfer. In the

Gaussian disorder model, the charge mobility in organic materials mainly depends on the variance of the state energy.[6] Here, we briefly describe the calculation of energetic disorder for organic materials. The environmental effect corresponds to the polarization effect, which can be explicitly considered as the intermolecular electrostatic interactions between a molecule and its environment and is reasonably represented by the distribution of the electrostatic potential (ESP) partial charges. The state energies can be determined by ab-initio quantum mechanical calculations, which are repeated for each molecule in a charge distribution within a cutoff distance self-consistently. Finally, the state energy differences are obtained for all neighboring pairs of molecules.

In order to quantify the energetic disorder involving the charge transfer, we used QuantumPatch implemented in Nanomatch software,[2,5,6] which employs Turbomole as the engine for the density functional theory (DFT) calculations. The level of B3LYP/def2SVP was chosen. We set the standard deviation of the excitation energies as 0.05 eV to reduce the computation time. The electronic couplings of the initial and final states during the charge transfer, denoted by J_{if}, were calculated for all dimers using QuantumPatch. The software computed the overlap of the HOMO orbitals of uncharged monomers for the hole transfer, and the overlap of the LUMO orbitals for the electron transfer. Similarly, Dexter couplings for the energy transfer were calculated according to the framework described.[1] The reorganization energy, λ_{if}, for charge transfer was obtained by calculating the optimized structure energy of the charged molecule, starting from the optimized geometry of a neutral molecule. The reorganization energy for energy transfer was set to 0.2 eV for all molecules in this study. We included the long-range energy transfer by Förster resonance energy transfer setting of the coupling as $J_{if} = \kappa\frac{|\mu_i||\mu_f|}{R^3}$, where μ_i and μ_f are the transition dipoles of the excitations, R is the distance between two molecules, and the orientation factor κ is set to 1/3.

The microscopic structure of organic materials is necessary for the calculation of material parameters such as the state energy disorder and electronic coupling. In this study, the morphologies of all layers in the OLED devices are obtained via deposition simulations based on the Monte Carlo method. The calculation of the energy is based on a classical model, where the intermolecular interactions involve potential energies depending on the dihedral angles, van der Waals (vdW) interactions, and electrostatic interactions. The potentials for dihedral rotations are determined though rotational scanning of the DFT energy with respect to all dihedral angles. The vdW interactions are described based on the Lennard–Jones potentials using the atomistic parameters compiled in Table S1, whereas the electrostatic interactions are calculated using the ESP partial charges by means of the Ewald summation method. The details of the deposition simulations can be referred.[7] For each layer and interface in our OLED device, the deposition of each molecule was performed with simulated annealing MC simulation by varying the temperature from 4000 K to 300 K in 2×10^5 MC steps; the process was repeated for N=2000 molecules. The configuration corresponding to the minimum energy was chosen from 32 independent runs for realizing the deposition of one molecule. The resultant microscopic structures successfully reproduced the orientational anisotropy of the organic layer, which is known to influence the outcoupling efficiency of the OLED device.[8] Using these morphologies, we obtained the energetic disorder $\sigma_{\Delta E}$ for each molecular species consitituting the device, as provided in Table S2. We also observed that the electronic couplings decrease exponentially with the intermolecular distance and vary substantially according to the relative orientation.[5] The superexchange coupling was included in the computation of the electronic coupling so that the charge transport in the host–dopant system could be described more precisely.[9]

Our model for the OLED device was defined as a cubic lattice with the lattice spacing of 1 nm in a lateral dimension of 40 nm×40 nm, considering the periodic boundary condition. We set the device structure of our model as anode / HTL (30 nm, BCFA) / HTL2 (10 nm, HT

series) / exciton blocking layer (5 nm/5nm, oCBP, mCPD) / EML (40 nm, oCBP:mCBP-2CN: PtON7-dtb:HCF or ν-DABNA 5:5:10%:1.5%) / HBL (10 nm, mCP-2CN) / ETL (31 nm, co-deposited NET series:NDN series) / cathode, yielding a total length of 131 nm. We omitted the p-doped hole injection layer and made the first HTL shorter than the experimental bottom-emission OLED device studied here. In order to mitigate the injection barrier generated from the pile up of injected carrier at the anode/HTL and ETL/cathode interfaces in our model device, we adjusted the work functions of the anode and cathode to –6.1 eV and –2.5 eV, respectively. The material parameters involving the charge transfer and excitonic processes are presented in Tables S2 and S3. In Table S3, the excitation energies and (non)radiative decay rates for oCBP, mCBP-2CN, PtON7-dtb, HCF, and ν-DABNA were determined experimentally, whereas those for other materials were generic. In the case of fluorescent molecules, the singlet (triplet) decays quickly (slowly). The maximum rate for singlet quenching, denoted by k_q^{singlet}, including singlet-polaron quenching (SPQ), singlet-triplet annihilation (STA), and singlet-singlet annihilation (SSA) are assumed to be fast at 10^{10} s^{-1}, whereas that for triplet quenching, k_q^{triplet}, such as triplet-polaron quenching (TPQ) and triplet–triplet annihilation (TTA) is 10^6 s^{-1}. k_q^{triplet} for the two hosts in the emission layer forming the exciplex, for which the triplet exciton dynamics is expected to be slower than that of the phosphorescent emitter, is set to 10^6 s^{-1}. The phosphorescent emitter has microsecond radiative triplet decay, k_q^{triplet}, set to 10^{10} s^{-1}. The maximum rate for quenching refers to the case where the distance between two excitons is 1 nm and the quenching rates decays according to r^{-6}.

We analyzed the carrier/exciton density and recombination rate for the configuration of particles in the emitting layer at the applied voltage of 6.55 V (electric field, 0.05 eV/nm). We obtained the average of 80,000–100,000 snapshots obtained from 100 trajectories run

independently for 10^8 MC steps, while discarding the transient behavior corresponding to the first half of each trajectory.

Table S1. Lennard–Jones potential parameters

Atom	σ (Å)	ϵ (kJ/mol)
C	3.56	0.1506
N	2.9	0.1601
O	2.85	0.2008
H	1.78	0.01912
Pt	3.5	0.06597
B	3.5	0.06597

Table S2. Energy parameters

Layer	Materials	IP (eV)[a]	EA (eV)	λ (eV)		$\sigma_{\Delta E}$ (eV)		σ_{model} (eV)[b]
				Hole	Electron	HOMO	LUMO	
HIL	BCFA	6.65	2.24	0.20	0.22	0.15	0.11	0.05
HTL	HT series	6.69	2.30	0.13	0.23	0.17	0.12	0.05
EBL	oCBP	6.53	2.03	0.08	0.43	0.08	0.09	0.05
	mCPD	6.79	1.98	0.10	0.64	0.15	0.21	0.10
EML	oCBP	6.53	2.03	0.08	0.43	0.18	0.18	0.15
	mCBP-2CN	6.89	2.11	0.06	0.43	0.18	0.21	0.15
	PtON7-dtb	5.93	2.01	0.20	0.44	0.16	0.15	0.1
	TBPDP	6.21	2.13	0.32	0.30	0.20	0.21	0.1
	ν-DABNA	5.89	1.50	0.17	0.22	0.15	0.17	0.1
HBL	mCP-2CN	7.02	2.15	0.10	0.21	0.22	0.22	0.17
ETL[c]		6.89	1.94	0.13	0.23	-	-	0.05

[a]AC3 HOMO + 0.6 eV except for HIL and HTL
[b]A simplified parameter set for the energetic disorder used for assigning IP and EA energies to the lattice sites in our model device.
[c]Arbitrary parameters are used due to the absence of molecular structure information.

Table S3. Excitonic parameters

Layer	Materials	S_1 (eV)	T_1 (eV)	$k_{\mathrm{r}}^{\mathrm{singlet}}$ (s^{-1})	$k_{\mathrm{nr}}^{\mathrm{singlet}}$ (s^{-1})	$k_{\mathrm{r}}^{\mathrm{triplet}}$ (s^{-1})	$k_{\mathrm{nr}}^{\mathrm{triplet}}$ (s^{-1})	k_{ISC} (s^{-1})	k_{RISC} (s^{-1})	Max. $k_{\mathrm{q}}^{\mathrm{singlet}}$ (s^{-1})	Max. $k_{\mathrm{q}}^{\mathrm{triplet}}$ (s^{-1})
HTL	BCFA			10^8	10^4	10^3	10^4	10^5	10^3	10^{10}	10^6
HTL2	HT series			10^8	10^4	10^3	10^4	10^5	10^3	10^{10}	10^6
EBL	oCBP	3.10	3.00	10^8	10^4	10^3	10^4	10^5	10^3	10^{10}	10^6
	mCPD			10^8	10^4	10^3	10^4	10^5	10^3	10^{10}	10^6
EML	oCBP	3.10	3.00	10^6	10^7	10^{-3}	10^4	10^5	10^3	10^5	10^5
	mCBP-2CN	2.93	2.81	10^6	10^7	10^{-3}	10^4	10^5	10^3	10^5	10^5
	PtON7-dtb	2.86	2.73	10^8	10^4	1.37×10^5	10^4	10^{14}	10^3	10^{10}	10^{10}
	TBPDP	2.63	1.70	1.32×10^8	9.18×10^6	10^{-3}	10^4	10^5	10^3	10^{10}	10^6
	ν-DABNA	2.66	2.64	9.45×10^7	1.69×10^6	10^{-3}	10^4	10^5	10^5	10^{10}	10^6
HBL	mCP-2CN	3.12	3.02	10^8	10^4	10^3	10^4	10^5	10^3	10^{10}	10^6
ETL				10^8	10^4	10^3	10^4	10^5	10^3	10^{10}	10^6

Table S4. Densities of hole, electron, triplet excitons, and recombination rates at oCBP, mCBP-2CN, PtON7-dtb, TBPDP, and ν-DABNA molecules in the emitting layers.

		**oCBP**	**mCBP-2CN**	**PtON7-dtb**	**TBPDP**	**ν-DABNA**
PtON7-dtb (10%)	Hole (10^{16} cm^{-3})	0.2	0.0	30.9	-	-
	Electron (10^{16} cm^{-3})	1.7	9.0		-	-
	Triplet Exciton (10^{16} cm^{-3})	0.01	2.7	1.9	-	-
	Recombination (10^{21} cm^{-3} s^{-1})	0.6	1.5	3.7	-	-
PtON7-dtb:TBPDP (10%:1.5%)	Hole (10^{16} cm^{-3})	0.2	0.0	30.6	0.1	-
	Electron (10^{16} cm^{-3})	1.5	10	0.001	0.003	-
	Triplet Exciton (10^{16} cm^{-3})	0.008	1.6	0.6	1.5	-
	Recombination (10^{21} cm^{-3} s^{-1})	0.6	1.5	3.7	0.05	-
PtON7-dtb: ν-DABNA (10%:1.5%)	Hole (10^{16} cm^{-3})	0.08	0.0	24.8	-	6.3
	Electron (10^{16} cm^{-3})	1.8	9.5	0.001	-	0.0
	Triplet Exciton (10^{16} cm^{-3})	0.005	1.8	0.7	-	0.6
	Recombination (10^{21} cm^{-3} s^{-1})	0.5	1.5	2.9	-	0.73
TBPDP (1.5%)	Hole (10^{16} cm^{-3})	17.5		-	13.5	-
	Electron (10^{16} cm^{-3})	1.9	9.0	-	0.01	-
	Triplet Exciton (10^{16} cm^{-3})	0.07	14.6	-	7.4	-
	Recombination (10^{21} cm^{-3} s^{-1})	3.8	2.8	-	1.3	-
ν-DABNA (1.5%)	Hole (10^{16} cm^{-3})	3.9	0.001	-	-	27.5
	Electron (10^{16} cm^{-3})	1.6	10.9	-	-	0.0
	Triplet Exciton (10^{16} cm^{-3})	0.04	6.2	-	-	1.9
	Recombination (10^{21} cm^{-3} s^{-1})	1.3	2.0	-	-	3.0

[a]Excitons are modeled as a localized state in a single molecule in the current model, where the feature of exciplex formation on host molecules is incorporated as a long lifetime of the singlet state.

Table S5. Summary of the state-of-the-art best reports on high-efficiency and long-lifetime deep-blue OLEDs with CIEy below 0.3

Emitter	V[a] (V)	CE_{max} /CE[a] (cd/A)	EQE_{max} /EQE[a] (%)	Lumi. (cd m^{-2})	CIE	LT50 (h)	Emitting Direction	Ref.
Graded $Ir(dmp)_3$ (tandem)	14.3	N.A.	N.A. / 18.0	1000	(0.15, 0.29)	~3500[c]	Bottom	10
Ir Complex: TBPDP	5.1	N.A.	10.5/10.5	1000	(0.14, 0.27)	~1500[c]	Bottom	11
$Ir(cb)_3$: t-DABNA	6.0	23.4 / 18.7	24.6 / 19.4	1000	(0.13, 0.12)	~17	Bottom	12
P4TCPhBN: t-DABNA	N.A	N.A.	32.5 / 23.2	1000	(0.13, 0.12)	~300[c]	Bottom	13
4TCzBN	6.0	N.A	16.2 / 5.5	1000	(0.16, 0.22)	~60[c]	Bottom	14
ν-DABNA	6.2	31.0 / 23.2	34.4 / 26.0	1000	(0.12, 0.11)	~1	Bottom	15
HDT-1: ν-DABNA	5.6	39 / 31	27 / 20	1000	(0.15, 0.20)	~500[c]	Bottom	16
HDT-1: ν-DABNA (tandem)	11.5	72 / 59	41 / 32	1000	(0.13, 0.16)	~800[c]	Bottom	16
PPCzTrz: ν-DABNA	6.4	39.7 / 28.9	33.0 / 25.2	1000	(0.13, 0.20)	151	Bottom	17
PCzTrz: ν-DABNA	6.7	35.5 / 24.3	33.5 / 23.8	1000	(0.12, 0.18)	113	Bottom	17
PPCzTrz: ν-DABNA	5.3	32.8 / 23.6	29.3 / 21.4	1000	(0.12, 0.09)	117	Top	17
PCzTrz: ν-DABNA	4.9	38.9 / 24.4	34.4 / 21.9	1000	(0.12, 0.09)	97	Top	17
PtON7-dtb:TBPDP	4.8	28.9 / 24.6	16.9 / 14.3	1000	(0.13, 0.27)	339	Bottom	This work
PtON7-dtb: ν-DABNA	4.7	32.0 / 25.1	32.2 / 25.4	1000	(0.11, 0.14)	117	Bottom	This work
PtON7-dtb: TBPDP	4.9	23.8 / 16.1	-	1000	(0.11, 0.13)	192	Top	This work
PtON7-dtb: ν-DABNA	4.5	48.9 / 37.8	-	1000	(0.12, 0.09)	254	Top	This work

[a]Values at a luminance of 1000 cdm^{-2}

[b]N.A. denotes that data was not assigned.

[c]Estimated from extrapolation

References

[1] F. Symalla, S. Heidrich, P. Friederich, T. Strunk, T. Neumann, D. Minami, D. Jeong, W. Wenzel, *Adv. Theory Simul.* **2020**, *3*, 1900222.

[2] Nanomatch.com (Nanomatch GmbH, Germany)

[3] R. A. Marcus, *Rev. Mod. Phys*. **1993**, **65**, 599.

[4] V. Rühle, A. Lukyanov, F. May, M. Schrader, T. Vehoff, J. Kirkpatrick, B. Baumeier, D. Andrienko, *J. Chem. Theory Comput.* **2011**, *7*, 3335-3345.

[5] P. Friederich, F. Symalla, V. Meded, T. Neumann, W. Wenzel, *J. Chem. Theory Comput.* **2014**, *10*, 3720-3725.

[6] H. Bässler, *Phys. Status Solidi B* **1993**, *175*, 15-56.

[7] T. Neumann, D. Danilov, C. Lennartz, W. Wenzel, *J. Comput. Chem.* **2013**, *34*, 2716-2725.

[8] J. S. Kim, D. Jeong, H. J. Bae, Y. Jung, S. Nam, J. W. Kim, S.-G. Ihn, J. Kim, W.-J. Son, H. Choi, S. Kim *Adv. Opt. Mater.* **2020**, *8*, 2001103.

[9] F. Symalla, P. Friederich, A. Massé, V. Meded, R. Coehoom, P. Bobbert, W. Wenzel, *Phys. Rev. Lett.* **2016**, *117*, 276803.

[10] Y. Zhang, J. Lee, S. R. Forrest, *Nat. Commun.* **2014**, 5, 5008.

[11] P. Heimel, A. Mondal, F. May, W. Kowalsky, C. Lennartz, D. Andrienko, R. Lovrincic, *Nat. Commun.* **2018**, 9, 4990.

[12] K. H. Lee, J. Y. Lee, *J. Mater. Chem. C.* **2019**, 7, 8562.

[13] D. Zhang, X. Song, A. J. Gillett, B. H. Drummond, S. T. E. Jones, G. Li, H. He, M. Cai, D. Credgington, L. Duan, *Adv. Mater.* **2020**, 32, 1908355.

[14] D. Zhang, M. Cai, Y. Zhang, D. Zhang, L. Duan, *Mater. Horiz.* **2016**, 3, 145.

[15] Y. Kondo, K. Yoshiura, S. Kitera, H. Nishi, S. Oda, H. Gotoh, Y. Sasada, M. Yanai, T. Hatakeyama, *Nat. Photon.* **2019**, 13, 678.

[16] C.-Y. Chan, M. Tanaka, Y.-T. Lee, Y.-W. Wong, H. Nakanotani, T. Hatakeyama, C. Adachi, *Nat. Photon.* **2021**, 15, 203.

[17] S. O. Jeon, K. H. Lee, J. S. Kim, S.-G. Ihn, Y. S. Chung, J. W. Kim, H. Lee, S. Kim, H. Choi, J. Y. Lee, *Nat. Photon.* **2021**, 15, 208.
